# Abdominal Obesity and Lung Cancer Risk: Systematic Review and Meta-Analysis of Prospective Studies

**DOI:** 10.3390/nu8120810

**Published:** 2016-12-15

**Authors:** Khemayanto Hidayat, Xuan Du, Guochong Chen, Minhua Shi, Bimin Shi

**Affiliations:** 1 Department of Endocrinology and Metabolism, the First Affiliated Hospital of Soochow University, Suzhou 215006, China; khemzie_khem@yahoo.com (K.H.); cathy-dx0630@163.com (X.D.); 2 Department of Nutrition and Food Hygiene, School of Public Health, Soochow University, Suzhou 215123, China; lsguorong@126.com; 3 Department of Respiratory Medicine, the Second Affiliated Hospital of Soochow University, Suzhou 215000, China; shiminhua@163.com

**Keywords:** abdominal obesity, central obesity, dose-response, lung cancer, waist circumference, waist to hip ratio

## Abstract

Several meta-analyses of observational studies have been performed to examine the association between general obesity, as measured by body mass index (BMI), and lung cancer. These meta-analyses suggest an inverse relation between high BMI and this cancer. In contrast to general obesity, abdominal obesity appears to play a role in the development of lung cancer. However, the association between abdominal obesity (as measured by waist circumference (WC) (BMI adjusted) and waist to hip ratio (WHR)) and lung cancer is not fully understood due to sparse available evidence regarding this association. PubMed and Web of Science databases were searched for studies assessing the association between abdominal obesity and lung cancer up to October 2016. The summary relative risks (RRs) with 95% confidence intervals (CIs) were calculated with a random-effects model. Six prospective cohort studies with 5827 lung cancer cases among 831,535 participants were included in our meta-analysis. Each 10 cm increase in WC and 0.1 unit increase in WHR were associated with 10% (RR 1.10; 95% CI 1.04, 1.17; *I*^2^ = 27.7%, *p*-heterogeneity = 0.198) and 5% (RR 1.05; 95% CI 1.00, 1.11; *I*^2^ = 25.2%, *p*-heterogeneity = 0.211) greater risks of lung cancer, respectively. According to smoking status, greater WHR was only positively associated with lung cancer among former smokers (RR 1.11; 95% CI 1.00, 1.23). In contrast, greater WC was associated with increased lung cancer risk among never smokers (RR 1.11; 95% CI 1.00, 1.23), former smokers (RR 1.12; 95% CI 1.03, 1.22) and current smokers (RR 1.16; 95% CI 1.08, 1.25). The summary RRs for highest versus lowest categories of WC and WHR were 1.32 (95% CI 1.13, 1.54; *I*^2^ = 18.2%, *p*-heterogeneity = 0.281) and 1.10 (95% CI 1.00, 1.23; *I*^2^ = 24.2%, *p*-heterogeneity = 0.211), respectively. In summary, abdominal obesity may play an important role in the development of lung cancer.

## 1. Introduction

Lung cancer is the most common cancer in the world with 1.8 million new cases diagnosed in 2012, accounting for 12.9% of total cancer incidences. In terms of mortality, 1.6 million deaths were caused by lung cancer in 2012, accounting for 19.4% of total cancer deaths [1]. There is emerging evidence that general obesity and/or abdominal obesity is associated with increased risk of certain types of cancers, including postmenopausal breast cancer, colorectal, endometrial, esophagus, kidney, pancreatic, thyroid, and gallbladder cancers [2,3,4,5,6,7,8,9,10,11]. However, evidence is less convincing and somewhat controversial for lung cancer. Several meta-analyses of observational studies have been performed to examine the association between general obesity, as measured by body mass index (BMI), and lung cancer. These meta-analyses suggest an inverse relation between high BMI and this cancer [12,13,14]. Cigarette smoking has been directly associated with increased lung cancer risk; thus, an inverse relationship between BMI and lung cancer may be a reflection of incomplete adjustment for the effects of cigarette smoking, as smoking habits may affect both body weight and body composition [15,16,17]. In contrast to general obesity, body fat distribution—particularly abdominal obesity—appears to play a role in the development of lung cancer [18,19,20,21]. Abdominal obesity is reflected by a higher waist to hip ratio (WHR) and a higher waist circumference (WC) relative to others with similar BMI [21]. Findings from several prospective cohort studies have found a positive association between lung cancer incidence and WHR and/or WC after adjustment for BMI [18,19,20,21]. While unadjusted WC reflects general obesity, only adjusted WC reflects abdominal obesity [21]. Furthermore, another study has also found a positive association between higher WC and lung cancer mortality [22]. Thus far, the association between abdominal obesity and lung cancer is not fully understood due to sparse available evidence regarding this association [18,19,20,21,23,24]. Given these considerations, we conducted the present meta-analysis of prospective studies with the following objectives: (1) provide insight into and robust evidence concerning the association between abdominal obesity and lung cancer by using published prospective data; and (2) investigate and quantify the potential dose-response relationship between abdominal adiposity measures and risk of lung cancer that has not been investigated before.

## 2. Materials and Methods

### 2.1. Search Strategy

This meta-analysis was planned, conducted, and reported according to “Meta-Analysis of Observational Studies in Epidemiology (MOOSE) group” guidelines [25]. PubMed and Web of Science databases were searched for studies assessing the association between abdominal obesity and lung cancer up to October 2016. The following search terms were employed to retrieve the relevant literature in the databases: (adiposity OR body size OR anthropometric OR abdominal obesity OR central obesity OR obese OR abdominal adiposity OR obesity OR body composition OR body fat distribution OR body fat patterning OR retroperitoneal fat OR visceral fat OR abdominal fat OR intra-abdominal fat OR waist to hip ratio OR waist hip ratio OR waist circumference OR girth circumference OR abdominal adiposity measures OR adiposity measures) AND (lung cancer OR cancer of lung OR lung carcinoma OR carcinoma of lung OR adenocarcinoma of the lung OR lung adenocarcinoma OR small cell lung cancer OR small cell lung carcinoma OR non-small cell lung cancer OR non-small cell lung carcinoma OR squamous cell lung cancer OR squamous cell lung carcinoma OR neoplasm of lung OR lung neoplasm OR lung tumor OR tumor of lung OR lung tumour OR tumour of lung OR NSCLC OR SCLC) AND (cohort OR prospective OR follow-up OR follow up OR observational study). The search strategy had no language, publication date, or publication type restriction. In addition, the reference lists of retrieved full publications were reviewed to complement the search and to identify relevant studies that were missed during the electronic database search.

### 2.2. Study Selection

To be included in this meta-analysis, the studies had to meet the following inclusion criteria: (a) the study had a prospective design (including prospective cohort study, nested case-control study, and case-cohort study); (b) examined the association between measures of abdominal obesity (WC and/or WHR) and risk of lung cancer; and (c) relative risks (RRs) or hazard ratios (HRs) or odds ratios (ORs) with 95% confidence intervals (CIs) were available. Accordingly, retrospective studies, or studies on lung cancer mortality or recurrence were excluded. If multiple publications from the same study were identified, the publication containing the largest number of cases and most detailed information (i.e., reporting data for subgroup or dose-response analyses) was selected.

### 2.3. Data Extraction and Quality Assessment

Using a standardized data-collection form, the following data were abstracted from each study: the first author’s last name, publication year, country, study population, duration of follow-up, number of participants, number of cases, ascertainment of adiposity, measures of abdominal adiposity, most fully adjusted risk estimates with their corresponding 95% CIs for each category of abdominal adiposity measures, adjustment for anthropometric variables, and adjustment for potential confounding factors. If multiple RRs of the association were available, we extracted RRs with their corresponding 95% CIs from the models that reflected the maximum extent of adjustment for potentially confounding variables, and from the models that further adjusted for anthropometric variables (e.g., BMI, height). When studies provided risk estimates according to smoking status, we extracted all of them and used the data in subgroup analysis. The study quality was assessed using the 9-star Newcastle-Ottawa Scale (NOS) [26], in which each study was judged based on the selection of the study groups (representativeness, selection of non-exposed cohort, ascertainment of exposure, no disease at start of study), the comparability of the groups, and three for the quality of the outcome (assessment of outcome, length of follow-up and adequacy of follow-up). Studies with NOS values of six or greater were considered moderate to high-quality studies and those with a NOS value of less than six were regarded low-quality studies. Two investigators (K.H. and X.D.) participated in literature search, study selection, data extraction, and quality assessment independently. Any discrepancies regarding inclusion were solved through group discussion, with input from the senior investigator (B.M.S.).

### 2.4. Statistical Analysis

RR was chosen as the common measure of association across this study, and HR was directly considered as RR. A DerSimonian and Laird random-effects model [27] was used to calculate the summary risk estimates. The degree of heterogeneity in the relationship between measures of abdominal obesity and lung cancer across studies was assessed using *Q* and *I*^2^ statistics. For the *Q* statistic, *p* < 0.1 was considered statistically significant; and for the *I*^2^ statistic, the following conventional cut-off points were used: <25% (low heterogeneity), 25%–50% (moderate heterogeneity) and >75% (severe heterogeneity). Both Begg’s rank correlation test and Egger’s linear regression test were performed to investigate potential publication bias [28]. If evidence of publication bias was observed, the trim and fill method was applied to correct the bias [29].

We only performed subgroup analysis according to smoking status (never smokers, former smokers and current smokers) due to the limited number of studies included in this meta-analysis. In addition, to investigate the impacts of individual studies on the overall results, we also performed a sensitivity analysis by omitting one study in each turn while pooling results from the remainder. We performed a linear dose-response analysis examining the association between measures of abdominal obesity and lung cancer risk according to the method proposed by Greenland and Longnecker [30] and Orsini et al. [31]. This method requires the number of cases and person-years and the risk estimates with their variance estimates for at least three quantitative abdominal adiposity measures categories. For the studies that did not provide the number of cases and/or person-years in each abdominal obesity measure category, we estimated these data from total number of cases and person-years. For each study, the median or mean level of abdominal obesity measures for each category was assigned to each corresponding risk estimate. When the median or mean abdominal obesity measures per category were not provided, we considered the midpoint of the upper and lower boundaries in each category as a new reference. If the highest or lowest category was open-ended, we assumed the width of the interval to be the same as in the closest category. Forest plots of the linear dose-response meta-analysis were presented for each 10 cm increase in WC and for 0.1 unit increase in WHR. All statistical analyses were performed using STATA software, version 11.0 (STATA Corp., College Station, TX, USA). All *p*-values were two-sided, and the level of significance was at <0.05, unless explicitly stated.

## 3. Results

### 3.1. Study Characteristics

**Figure 1:**
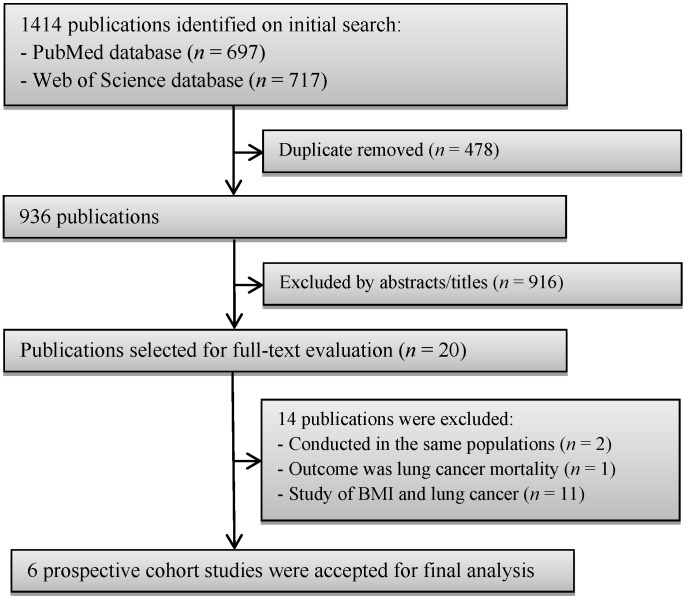
Flow chart of study selection.

A flow chart of study selection, including reasons for exclusion, is presented in Figure 1. During the initial search, we briefly identified 1414 articles from PubMed and Web of Science databases; most were excluded because they were retrospective studies or because the exposure or outcome was not relevant to our analysis, leaving 20 potentially eligible articles for full-text review. After careful review, 14 articles were further excluded; among excluded articles, two articles were conducted in the same populations [15,32], one article examined central obesity and lung cancer mortality [22], and the remaining 11 studies were excluded because the risk estimate for the association of interest was not available. Finally, six prospective cohort studies [18,19,20,21,23,24] were included into our final analysis. The characteristics of the included studies are summarized and listed in Table 1. These studies were published between 2002 and 2016. All of the included studies had a prospective cohort design. A total of 5827 lung cancer cases were diagnosed among 831,535 participants. One prospective cohort study was conducted in China [24], one in European countries [21], and the remaining four in the USA [18,19,20,23]. Regarding the sex of the participants, four [18,19,23,24] studies evaluated only women, and the remaining two [20,21] included both sexes. The length of follow-up ranged from seven to 15.1 years. Individual studies adjusted for a wide range of potential confounding factors, such as age, physical activity, and smoking. The details of quality assessment according to the nine-star NOS are presented in the online Supplementary Materials Table S1. All studies were given scores of ≥7.

### 3.2. WC and Lung Cancer

**Figure 2:**
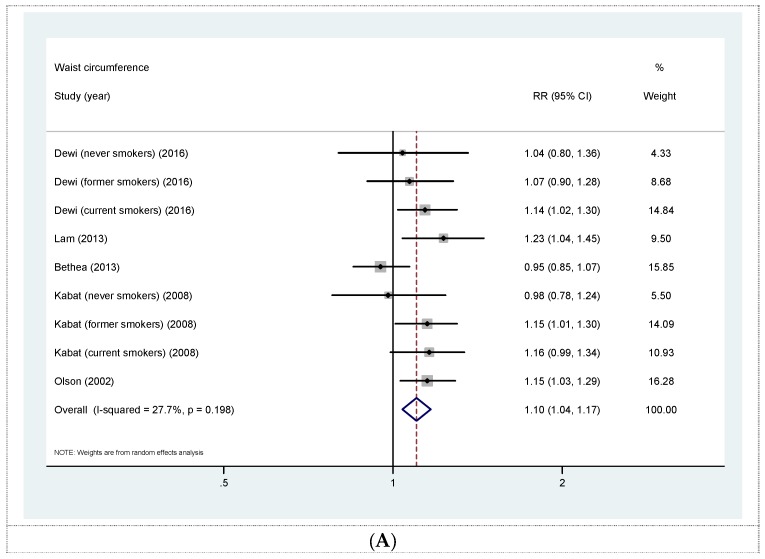

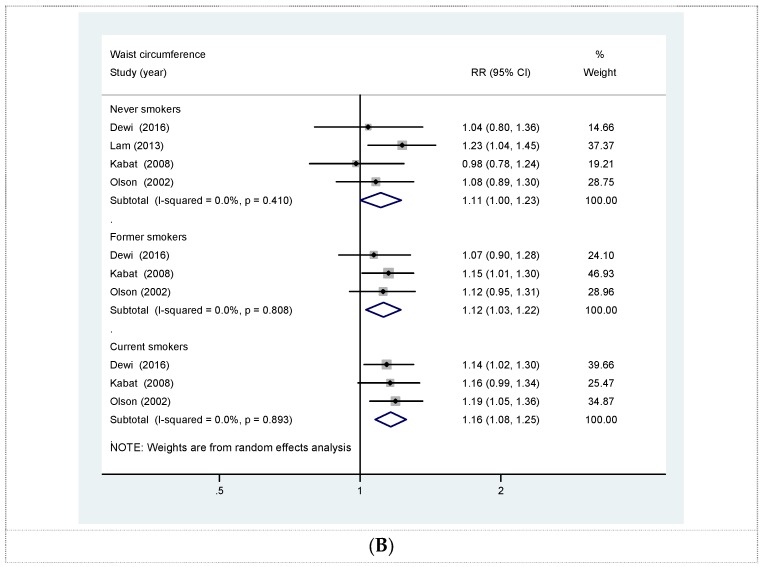
(**A**) Forest plot for linear dose-response analysis on waist circumference and lung cancer risk, per 10 cm increase. All risk estimates for waist circumference were additionally adjusted for body mass index (BMI); (**B**) forest plot for linear dose-response analysis on waist circumference and lung cancer risk stratified by smoking status. CI confidence interval; RR relative risk.

Five prospective cohort studies [18,19,20,21,23] were eligible for the analysis of WC and risk of lung cancer. All studies concerning this association were further adjusted for BMI. The summary RR for a 10 cm increase in WC was 1.10 (95% CI 1.04, 1.17) with moderate heterogeneity (*I*^2^ = 27.7%, *p* = 0.198) (Figure 2A). No evidence of publication bias was observed across studies (Begg, *p* = 0.404; Egger, *p* = 0.842). The summary RRs according to smoking status were 1.11 (95% CI 1.00, 1.23) for never smokers, 1.12 (95% CI 1.03, 1.22) for former smokers, and 1.16 (95% CI 1.08, 1.25) for current smokers (Figure 2B). Sensitivity analysis investigating the influence of a single study on the overall risk estimate by omitting one study at each turn yielded a range of RRs from 1.09 (95% CI 1.01, 1.18) to 1.13 (95% CI 1.08, 1.19). Furthermore, the summary RR for the highest versus lowest categories of WC was 1.32 (95% CI 1.13, 1.54) with low heterogeneity (*I*^2^ = 18.2%, *p* = 0.281) (Online Supplementary Materials Figure S1).

### 3.3. WHR and Lung Cancer

**Figure 3:**
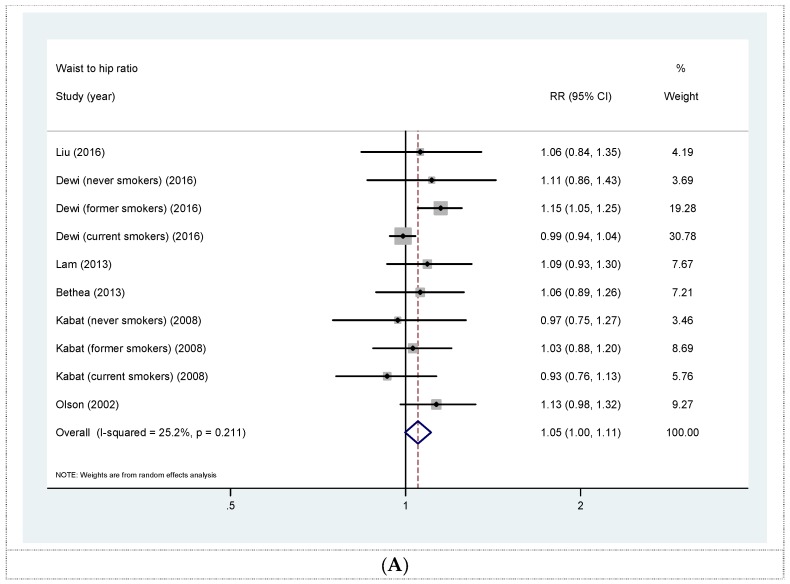

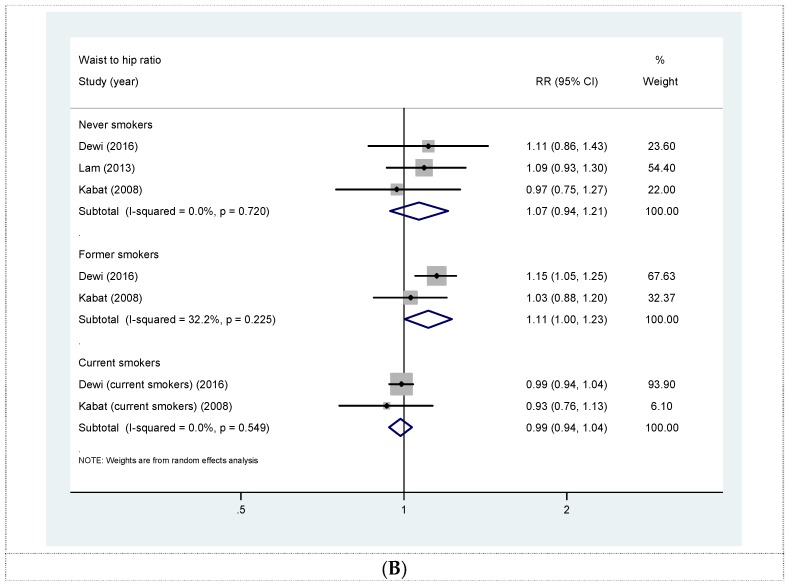
(**A**) Forest plot for linear dose-response analysis on waist to hip ratio and lung cancer risk, per 0.1 unit increase; (**B**) forest plot for linear dose-response analysis on waist to hip ratio and lung cancer risk stratified by smoking status. CI confidence interval; RR relative risk.

Six prospective cohort studies [18,19,20,21,23,24] were eligible for the analysis of WHR and risk of lung cancer. The summary RR for a 0.1 unit increase in WHR was 1.05 (95% CI 1.00, 1.11) with moderate heterogeneity (*I*^2^ = 25.2%, *p* = 0.211) (Figure 3A). No evidence of publication bias was observed across studies (Begg, *p* = 0.851; Egger, *p* = 0.962). The summary RRs according to smoking status were 1.07 (95% CI 0.94, 1.21) for never smokers, 1.11 (95% CI 1.00, 1.23) for former smokers, and 0.99 (95% CI 0.94, 1.04) for current smokers (Figure 3B). In a sensitivity analyses in which we omitted one study at a time, the overall association became slightly attenuated (RR 1.05, 95% CI 0.98, 1.12) by excluding the study by Dewi et al. [21]. In addition, the summary RR for the highest versus lowest categories of WC was 1.10 (95% CI 1.00, 1.23) with low heterogeneity (*I*^2^ = 24.2%, *p* = 0.211) (Online Supplementary Materials Figure S2).

## 4. Discussion

To our knowledge, the present meta-analysis is the first quantitative review of prospective studies concerning the association between abdominal obesity and lung cancer risk. Our dose-response analysis revealed that each 10 cm increase in WC and 0.1 unit increase in WHR were associated with 10% and 5% greater risks of lung cancer, respectively. In contrast, observational studies have reported a consistent inverse relationship between general obesity, as measured by BMI, and lung cancer [12,13,14]. Thus, our findings suggest that an excess of abdominal adipose tissue, but not overall body fatness, may be a better predictor of lung cancer. Although limited, our findings may have significant public health and clinical implications because WC (BMI adjusted) and to a lesser extent WHR may represent a better predictor for lung cancer than BMI. In that regard, obtaining a WC and WHR measurement instead of BMI in individuals already at increased risk for lung cancer (e.g., in current smokers and former smokers) may provide essential information that might not be delivered by BMI because smokers tend to have greater abdominal adiposity than nonsmokers do with similar BMI [33,34,35].

The exact mechanisms for the association between abdominal obesity and lung cancer remain poorly understood. One speculative biological mechanism for the contrary associations of abdominal obesity and general obesity to lung cancer may involve complex biologic pathways, such as hyperinsulinemia, decreased levels of sex hormone binding globulin (SBHG), and increased levels of unbound androgens and estrogens. All of these biologic pathways are more strongly related to abdominal fatness than to body fatness [15,18,20,36,37,38]. Moreover, several in vitro studies have shown that small-cell lung cancer and non-small-cell lung cancer respond to insulin-like growth factors I (IGF-I) [39,40] and that lung cancer cells contain receptors for steroid hormones, including estrogens and androgens [41,42,43]. Nevertheless, the biologic mechanism underlying the association between abdominal obesity and lung cancer warrants further research.

Furthermore, there are several reasonable explanations for the contrary relationships of abdominal obesity and general obesity to lung cancer in regard to the residual confounding by smoking. The interrelations between general and abdominal obesity measures and smoking are complex and are subject to change over time. Smoking is a well-established risk factor for lung cancer [44], and is also inversely associated with body weight [45,46]. Current smokers are associated with both lower BMI [35] and increased risk of lung cancer. By comparison, current smokers are associated with more visceral adipose tissue accumulation [22], and it is known that smoking cessation is associated with increased body weight and WC [18,22]. If this is the case, limiting the investigation to participants who never smoked may help to overcome the issue of residual confounding by smoking in regard to both general obesity and abdominal obesity.

A dose-response meta-analysis by Duan et al. [14] did not find inverse association of high BMI and lung cancer when restricting analysis to non-smokers. Thus far, only a few prospective studies have examined the association between abdominal obesity and risk of lung cancer among never smokers [18,19,20,21]. Olson et al. [18], Kabat et al. [19], and Dewi et al. [21] found WC was positively associated with lung cancer among smokers, but not among never smokers. All of these studies found that WC was positively associated with risk of lung cancer in smokers, but only after adjustment for BMI. Furthermore, in the largest prospective study of never smoking lung cancer to date, Lam et al. [20] also observed a significant positive association between WC, conditional on BMI, and lung cancer risk; this finding suggests that the observed positive associations from previous studies were not merely due to residual confounding by smoking. Nevertheless, data on an association between abdominal obesity and risk of non-smoking lung cancer remains inconclusive. Therefore, further large prospective studies conducted on never smokers are needed to eliminate residual confounding by cigarette smoking.

Our stratified analysis according to smoking status revealed that higher WC was associated with 11% increased risk of lung cancer in never smokers, whereas no association was observed for WHR. As expected, greater WC was also positively associated with lung cancer among former smokers and current smokers. Regarding WHR, we observed a positive association among former smokers, but not among current smokers. These findings were somewhat surprising and challenging to explain since both WC (BMI adjusted) and WHR are measures of abdominal obesity. There are several explanations for the discrepancies between measures of abdominal obesity. First, WC (BMI adjusted) may be a better predictor of abdominal obesity than WHR [47,48]. Second, the null association of lung cancer and WHR among never smokers and current smokers may be because WHR are difficult to interpret since participants could have low abdominal fat and abundant gluteal fat or vice versa; although not for all studies, higher fat deposition in hip region (gluteofemoral fat) has been shown to be inversely associated with lung cancer [20]. Thus, this inverse association may weaken the overall positive association. Third, the observed positive association between greater WHR and lung cancer among former smokers is reasonable, since smoking cessation is associated with increased WC [22] and further corroborated the association. Finally, given a lower number of studies on WHR that further stratified for smoking status, as compared to studies on WC, the observed null association could be due to lack of statistical power to detect the true effect. Nevertheless, both WC (BMI adjusted) and WHR are both crude measures of abdominal obesity and cannot distinguish between subcutaneous fat and visceral fat. Therefore, future studies with advanced imaging techniques (i.e., magnetic resonance imaging, computed tomography) are warranted to confirm these findings.

As mentioned earlier, the findings from several prospective cohort studies have found a positive association between lung cancer incidence and WC, but only after further adjustment for BMI [18,19,20,21]. In contrast to unadjusted WC, which is related with overall body fat, adjusted WC is associated with having more abdominal and visceral adipose tissue than others do with similar BMI and height [21,49]. However, because keeping BMI and height constant reflects keeping total body mass constant, an increase in abdominal adipose tissue must be accompanied by a decrease in other parts of the body, for example lean mass reduction or gluteofemoral fat [21,50]. Furthermore, lean mass reduction may be associated with preclinical lung cancer, which could also explain the positive associations from these studies [21].

Finally, it is worth taking into consideration that abdominal obesity and cancer share many risk factors, such as smoking, physical inactivity, and poor diet [51,52]. For example, findings from our stratified analysis revealed that higher WC has the strongest effect in current smokers than other groups. It is possible that higher WC among smokers is a consequence of greater levels of smoking or other poor lifestyle habits (e.g., lack of physical activity, unhealthy diet) that may contribute to lung carcinogenesis. Given these considerations, the observed association between abdominal obesity and lung cancer may be partly due to similar confounding factors shared by both conditions. Further clarification for the issue of whether abdominal obesity itself is associated with increased risk of lung cancer, rather than a proxy for another cancer risk factor, is needed.

### Strengths and Limitations

This meta-analysis has several strengths, including incorporated evidence and relevant studies to the date. Because results from individual studies often had insufficient statistical power, the enlarged sample size from this present meta-analysis may enhance the power to detect a significant association and provide more precise estimates of the effects. All of the included studies have long follow-up durations and have a prospective nature that thereby reduced the likelihood of potential biases (e.g., recall and selection biases). Furthermore, given the considerably heterogeneous categories of abdominal obesity measures among included studies, a dose-response meta-analysis is necessary since it provides better precision of risk estimates than merely conducting high versus low analysis.

There are several limitations in the present meta-analysis that should be acknowledged. First, almost all of the included studies were conducted in women. Thus, our findings might not apply to men. Unfortunately, we are unable to perform subgroup analysis according to sex of the participants in order to clarify whether the differences in body fat distribution between men and women may influence the overall positive association between abdominal obesity and risk of lung cancer. Further studies focusing on the influence of sex differences on this association are needed to address this issue. Second, although individual studies have considered a wide range of potential confounders in their analyses, the potential impacts of residual/unknown confounding factors on our findings cannot be completely excluded; for example, confounding from smoking may have contributed to observed positive association of abdominal obesity and lung cancer. Third, our analysis was limited by the number of included studies, which limits our ability to perform subgroup analyses (e.g., according to lung cancer subtypes, sex). Fourth, the possible errors in WC and WHR measurements obtained by self-report and self-measurement have led to overestimation or underestimation of the true association between abdominal obesity and lung cancer. Fifth, the possible dose-response meta-analysis measurement error should also be acknowledged, as it requires assumptions such as extrapolating the width of the open-ended lowest and highest boundaries from the closest category and assigning the midpoint of each category of abdominal obesity measures to corresponding relative risk. Sixth, because the findings of the current meta-analysis were mainly based on data from studies conducted in Western populations, additional research in other populations is warranted to generalize the findings. Finally, given all of these limitations, the results from our meta-analysis should always be treated with caution. 

## 5. Conclusions

In summary, abdominal obesity may play an important role in the development of lung cancer. Further large prospective studies conducted in never smokers and both sexes are needed to extend and confirm our findings.

## Figures and Tables

**Table 1:** Prospective studies of abdominal obesity and lung cancer. All risk estimates for waist circumference were additionally adjusted for body mass index (BMI). All of the risk estimates that are presented below are the ones that we used for the present meta-analysis.

References (Country)	Study Population (Age)	Duration of Follow-Up (Years)	Sample Size (Lung Cancer Cases)	Ascertainment of Adiposity	Measure of Adiposity	Categories, Highest vs. Lowest (Measurement Unit)	Adjusted RR (95% CI)	Adjustment for Anthropometric Variables	Adjustment for Confounders
Olson et al. 2002 (USA) [18]	Older women (55–69 years)	13	38,006 (596)	Self-measured	WC	>99.0 cm vs. ≤75.56 cm	All: 1.76 (1.14, 2.73); never smokers: 1.43 (0.69, 2.97); former smokers: 1.62 (0.85, 3.09); current smokers: 1.83 (1.11, 3.01)	BMI, BMI at age 18 years, and height	Age, pack-years of smoking, smoking status, physical activity score, educational level, and beer consumption
					WHR	>0.90 vs. ≤0.76	1.29 (0.96, 1.75)		
Kabat et al. 2008 (USA) [19]	Postmenopausal women (50–79 years)	8	161,809 (1365)	Trained	WC	≥97.6 cm vs. <74.6 cm	Never smokers: 1.01 (0.45, 2.28); former smokers ^a^: 1.50 (0.98, 2.31); current smokers ^b^: 1.56 (0.91, 2.69)	Height and BMI ^1^	Age, education, ethnicity, use of HRT, intakes of total fat, fruits, vegetables, alcohol, and total calories, physical activity, and study
					WHR	≥0.87 vs. <0.75	Never smokers: 1.01 (0.64, 1.66); former smokers ^a^: 1.02 (0.77, 1.35); current smokers ^b^: 0.89 (0.62, 1.27)		
Bethea et al. 2013 (USA) [23]	African American women (21–69 years)	7	56,944 (323)	Self-measured	WC	>93.9 cm vs. <71.1 cm	0.85 (0.54, 1.35)	BMI	Age, education, physical activity, alcohol consumption, parity, age at first birth, family history of lung cancer, geographic region, and pack-years of smoking
					WHR	>0.87 vs. <0.71	1.27 (0.86, 1.87)		
Lam et al. 2013 (USA) [20]	Never-smokers (50–71 years)	11	158,415 (532)	Self-measured	WC	Men: 110.5 cm vs. 86.4 cm; women: 99.1 cm vs. 70.6 cm	1.75 (1.09, 2.79)	BMI and hip circumference ^1^	Age, education, ethnicity, alcohol consumption, vigorous physical activity, physical activity at work, and total caloric intake
					WHR	Men: 1.02 vs. 0.88; women: 0.90 vs. 0.73	1.22 (0.83, 1.81)		
Dewi et al. 2016 (European countries) [21]	Men and women (30–70 years)	11	348,108 (2400)	Trained	WC	Men: ≥102 cm vs. <94 cm; women: ≥88 cm vs. <80 cm	Never smokers: 0.95 (0.54, 1.65); former smokers: 1.15 (0.80, 1.63); current smokers: 1.38 (1.10, 1.72)	Height and BMI ^1^	The duration of smoking, the lifetime number of cigarettes smoked, the number of cigarettes smoked at baseline, educational level, physical activity level, fruit consumption, vegetable consumption, meat consumption, fat intake, and energy intake
					WHR	Men: >1.00 vs. <0.95; women: >0.85 vs. <0.80	Never smokers: 0.76 (0.1, 1.15); former smokers: 1.44 (1.14, 1.82); current smokers: 0.98 (0.85, 1.12)		
Liu et al. 2016 (China) [24]	Shanghai women (40–70 years)	15.1	68,253 (611)	Trained	WHR	>0.85 vs. ≤0.77	1.03 (0.77, 1.37)	BMI	Education, total energy intake, total vegetable and fruit intake, total meat intake, leisure-time physical activity, alcohol consumption, menopausal status, spouse smoking exposure, parity, and family history of cancer

BMI: body mass index; CI: confidence interval; HRT: hormone replacement therapy; RR relative risk; WC: waist circumference; WHR: waist to hip ratio. ^a^ Additionally adjusted for pack-years and age at quitting smoking; ^b^ Additionally adjusted for pack-years of smoking; ^1^ Only for waist circumference.

## Supplementary Materials

The following are available online at http://www.mdpi.com/2072-6643/8/12/810/s1, Figure S1. (1) Forest plot of abdominal obesity and risk of lung cancer for the highest versus the lowest categories of waist circumference. All risk estimates for waist circumference were additionally adjusted for body mass index (BMI); (2) forest plot of abdominal obesity and risk of lung cancer for the highest versus the lowest categories of waist to hip ratio. CI confidence interval; RR relative risk. Table S1. Quality assessment according to the nine-star Newcastle-Ottawa Scale (NOS) ^a^.
