# Supplementary material for: Abdominal Obesity and Lung Cancer Risk: Systematic Review and Meta-Analysis of Prospective Studies
Source: Nutrients. 2016 Dec 15;8(12):810. doi: 10.3390/nu8120810 (PMC5188465; doi:10.3390/nu8120810)
Supplement: Supplementary file 1 [file nutrients-08-00810-s001.docx]

Supplementary Materials: Abdominal Obesity and Lung Cancer Risk: Systematic Review and Meta-Analysis of Prospective Studies

Khemayanto Hidayat, Xuan Du, Guochong Chen, Minhua Shi and Bimin Shi

**Table S1.** Quality assessment according to the nine-star Newcastle-Ottawa Scale (NOS) ^a^.

| **Study** | **Selection** | | | | **Comparability** | **Outcome** | | | **Total Stars** |
| --- | --- | --- | --- | --- | --- | --- | --- | --- | --- |
|  | **Representativeness of Exposed Cohort** | **Selection of the Non-Exposed Cohort** | **Ascertainment of Exposure** | **Demonstration That Outcome of Interest Was Not Present at Start of Study** | **Comparability of Cohorts on the Basis of the Design or Analysis** | **Assessment of Outcome** | **Was Followed Up Long Enough for Outcomes to Occur** | **Adequacy of Follow Up of Cohorts** |  |
| Olson, 2002 | 0 | 1 | 1 | 1 | 2 | 1 | 1 | 0 | 7 |
| Kabat, 2008 | 0 | 1 | 1 | 1 | 2 | 1 | 1 | 0 | 7 |
| Bethea, 2013 | 0 | 1 | 1 | 1 | 2 | 1 | 1 | 0 | 7 |
| Lam, 2013 | 0 | 1 | 1 | 1 | 2 | 1 | 1 | 0 | 7 |
| Dewi, 2016 | 1 | 1 | 1 | 1 | 2 | 1 | 1 | 0 | 8 |
| Liu, 2016 | 0 | 1 | 1 | 1 | 2 | 1 | 1 | 0 | 7 |

^a^ A study can be awarded a maximum of one star for each numbered item within the Selection and Outcome categories and a maximum of two stars for Comparability.

|  |  |
| --- | --- |
| (**1**) | (**2**) |

**Figure S1.** (**1**) Forest plot of abdominal obesity and risk of lung cancer for the highest versus the lowest categories of waist circumference. All risk estimates for waist circumference were additionally adjusted for body mass index (BMI); (**2**) forest plot of abdominal obesity and risk of lung cancer for the highest versus the lowest categories of waist to hip ratio. CI confidence interval; RR relative risk.
